# Simulation Study of 4H-SiC Trench Insulated Gate Bipolar Transistor with Low Turn-Off Loss

**DOI:** 10.3390/mi10120815

**Published:** 2019-11-26

**Authors:** Hong-kai Mao, Ying Wang, Xue Wu, Fang-wen Su

**Affiliations:** 1 The Key Laboratory of RF Circuits and Systems, Ministry of Education, Hangzhou Dianzi University, Hangzhou 310018, China; 171040042@hdu.edu.cn (H.-k.M.); 171040083@hdu.edu.cn (F.-w.S.); 2 National Key Laboratory of Analog Integrated Circuits, Chongqing 400060, China; xuew86@yeah.net

**Keywords:** 4H-SiC, turn-off loss, ON-state voltage, breakdown voltage (BV), IGBT

## Abstract

In this work, an insulated gate bipolar transistor (IGBT) is proposed that introduces a portion of the p-polySi/p-SiC heterojunction on the collector side to reduce the tail current during device turn-offs. By adjusting the doping concentration on both sides of the heterojunction, the turn-off loss is further reduced without sacrificing other characteristics of the device. The electrical characteristics of the device were simulated through the Silvaco ATLAS 2D simulation tool and compared with the traditional structure to verify the design idea. The simulation results show that, compared with the traditional structure, the turn-off loss of the proposed structure was reduced by 58.4%, the breakdown voltage increased by 13.3%, and the forward characteristics sacrificed 8.3%.

## 1. Introduction

In recent years, with the development of semiconductor technology, market demand and power electronics have undergone great changes [1,2,3,4,5]. The market not only requires semiconductor devices that work properly in harsh environments, but devices with small sizes and high integration. Since Si-based devices have reached their material limits, third-generation semiconductor materials represented by SiC are widely used in device design.

There are two mainstreams processing methods for a 4H-SiC insulated gate bipolar transistor (IGBT) at present: one is to make a good compromise between the forward and off characteristics of the device by properly setting the structural parameters of the device such as the n buffer’s thickness and doping parameters [6], the minority carrier lifetime in the n^-^ drift region [7,8], and thickness and doping parameters of the CSL (carrier storage layer) [9,10]; the other is by considering the process conditions and designing a special device structure that can improve by affecting certain characteristics, such as an anode short circuit IGBT [11,12], a super junction IGBT [13], or a collector trench IGBT (CT-IGBT) with an electronic extraction channel [14]. By analyzing the previous research, it can be observed that researchers have been mainly concerned with the compromise between the on-state and breakdown characteristics of IGBT devices, and that relatively little research has been made into the dynamic conversion characteristics of the device.

In this paper, we propose an improved structure of introducing a partial p-polySi/p-SiC heterojunction on the collector side, which is named H-IGBT. By adjusting the doping concentration on both sides of the heterojunction, it is ensured that the heterojunction contributes to electron bleed under the premise of not affecting the forward characteristics, and that the tail current of the device is greatly reduced and turn-off loss is improved.

## 2. Fabrication Procedure and Parameters

### 2.1. Device Structure

**Figure 1:**
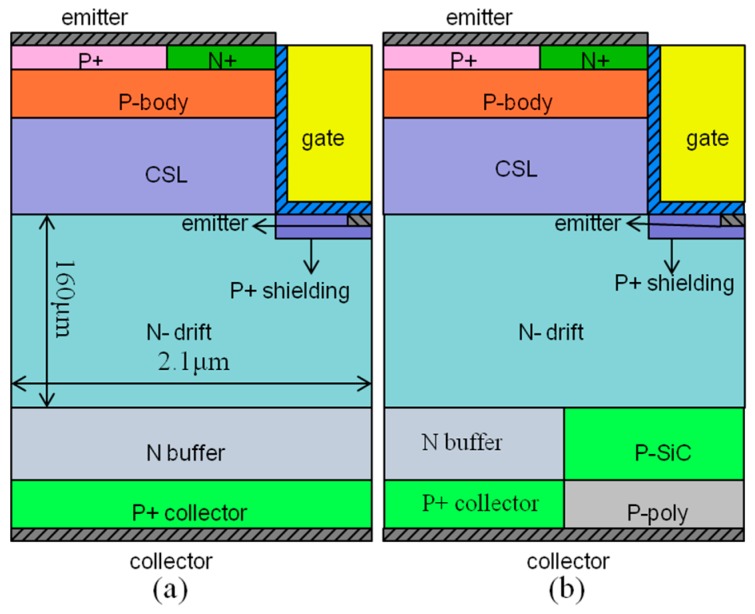
The schematic cross-sectional views of the (**a**) conventional insulated gate bipolar transistor (C-IGBT) and (**b**) heterojunction insulated gate bipolar transistor (H-IGBT).

Figure 1 is a schematic cross-sectional view showing the half-cell structure of a conventional IGBT device (C-IGBT) and the proposed structure of the H-IGBT, respectively. In order to verify the characteristic advantages of the proposed structure, we simulated the electrical characteristics of the device using Silvaco ATLAS two-dimensional simulation software. In the simulation process, we first design the basic structure of a breakdown voltage of 15 kV as the reference structure [15,16], and then apply the design idea to the new structure while keeping most of the parameters unchanged. The relevant parameters of the two structures during the simulation process are listed in Table 1 [17,18]. In this simulation, the carrier lifetime in the n^-^ drift region is 1 μs, and the carrier lifetime of the n buffer is 0.1 μs.

### 2.2. Proposed Fabrication Procedure

**Figure 2:**
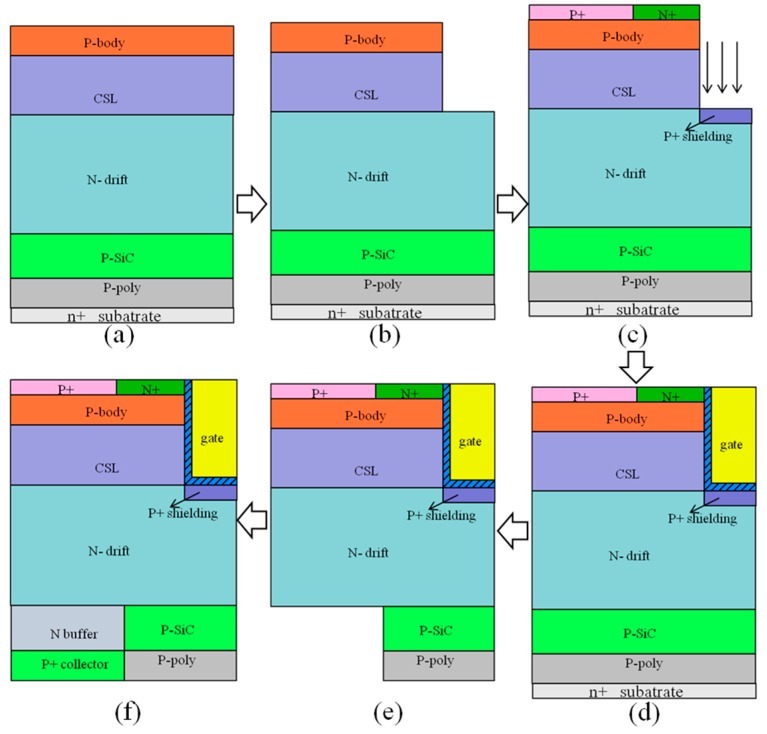
H-IGBT feasibility manufacturing process. (**a**) Epitaxial layers. (**b**) Forming a trench region by dry etching. (**c**) Source region and shield layer formed by ion implantation. (**d**) Forming a gate by growing an oxide layer and filling the polysilicon. (**e**) Forming a normal PN junction region by dry etching. (**f**) Forming a normal PN junction portion by epitaxy.

Since the relevant flow test work has not been performed, Figure 2 shows the feasibility manufacturing process of H-IGBT. A p-poly layer, a p-SiC layer, a N^-^ drift layer, a CSL layer, a p-body layer, and so on are sequentially grown on the N^+^ substrate [19,20], as shown in Figure 2a. Then, dry etching [21,22] is used to form gate trench regions, as shown in Figure 2b. The P^+^ shield, P^+^ source region, and N^+^ source regions are formed by ion implantation [23,24], as shown in Figure 2c. The gate oxide layer is thermally grown in dry O_2_ [25,26,27] and the trench regions are filled with polysilicon [28], as shown in Figure 2d. The substrate is removed and backside p-polySi and p-SiC epitaxial layers are dry-etched, as shown in Figure 2e. Epitaxial growing n buffer and P^+^ collector in the etched portion are shown in Figure 2f, respectively. The reason why the gate oxide layer is grown under dry oxygen conditions is to avoid the problem of the oxide layer at the bottom of the trench being too thin under thermal oxidation conditions. Finally, all electrodes are metalized, including emitter, gate, and collector.

In the manufacturing process of the device, it should be noted that in order to obtain a high quality gate oxide layer, a dry oxygen process is selected. In actual production, it is often necessary to comprehensively consider the effects of film formation quality and production efficiency using a dry oxygen-wet oxygen-dry oxygen process; meanwhile, because SiC materials are special, their hardness is relatively large, so the etching is very difficult. SiO_2_ can be used as a mask and etched by an inductively coupled plasma (ICP) etching method containing SF_6_. The specific etching scheme can use a combination of SF_6_/O_2_/A_r_ gases, with a flow rate of 4.2/8.4/28 sccm, a pressure and temperature of 0.4 Pa and 80 °C, respectively, an ICP power of 500 W and a bias power fixed at 15 W [29]. Attention should be paid to the formation of micro-grooves throughout the ICP etching process, which can cause an electric field concentration effect that in turn reduces the breakdown voltage of the device. After the etching, the surface of the trench will inevitably appear rough, which can be improved by the subsequent high temperature annealing process.

## 3. Simulation Results and Discussion

The material parameters and simulation models used in the simulation process are based on previous studies. Since these parameters have been widely used in the simulation of 4H-SiC IGBT, and the simulation results have been proved by experiments, these parameters and models have also been applied to this simulation. The models used in the simulation mainly include an energy band narrowing model (BGN), a parallel electric-field-dependent model (FLDMOB), a Fermi model, a concentration-dependent mobility model (CONMOB), and recombination models (Schockley-read-hall, AUGER) [30,31].

### 3.1. Forward Characteristics

**Figure 3:**
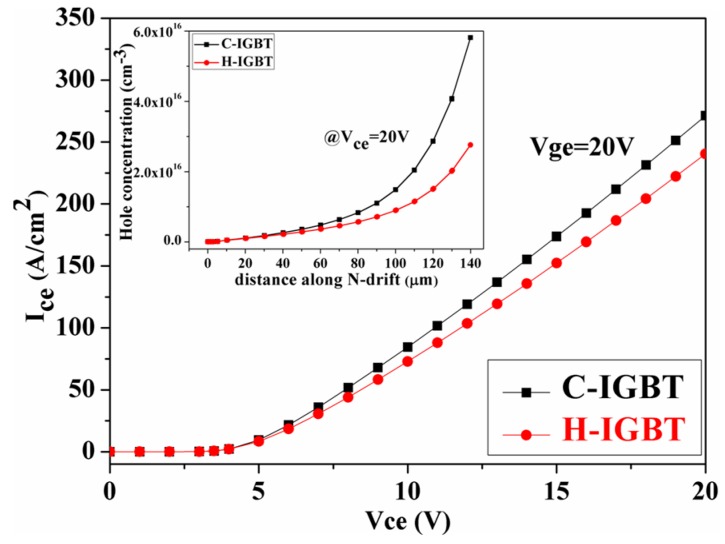
Forward I-V characteristics of C-IGBT and H-IGBT and the concentration distribution of holes in the entire drift region (inset) at x = 1.1μm.

Figure 3 shows the forward I-V characteristic curves of C-IGBT and H-IGBT and the hole concentration distribution through the drift region. It can be seen from the figure that the on-state characteristics of H-IGBT are slightly lower than C-IGBT. When V_ge_ = 20 V and I_ce_ = 100 A/cm^2^, the on-state voltage drops of the two structures are 11.7 and 10.8 V, respectively. After data analysis, it can be concluded that the conduction voltage drop of the improved structure is increased by 8.3% compared to the conventional structure. From the heterojunction band diagram, the p-polySi/p-SiC junction contributes to hole injection, but the number of holes injected into the drift region is controlled by the upper PN junction, at which point the bias voltage of the PN junction above the heterojunction is small. As such, the total hole injection efficiency is lower than that of the left half of the PN junction and the forward characteristics of the C-IGBT are superior to those of the H-IGBT. The hole distribution of the drift region in the figure further confirms the above explanation.

### 3.2. Breakdown Characteristics

**Figure 4:**
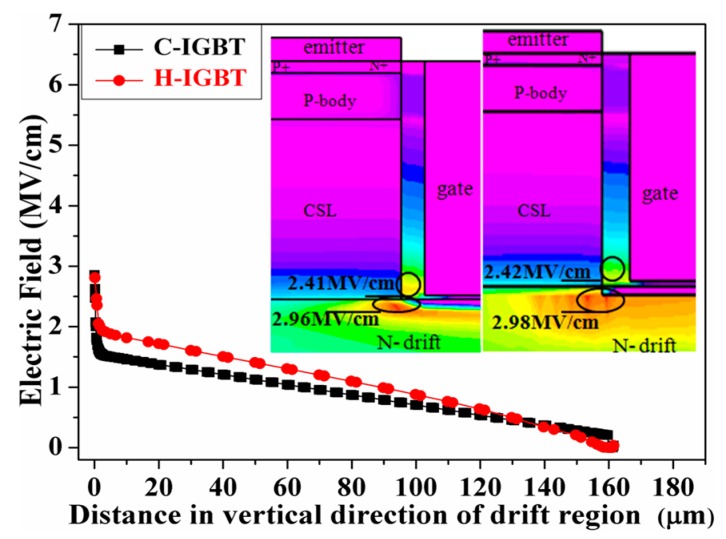
The electric field distribution of the C-IGBT and H-IGBT at the time of breakdown and the electric field cut of the entire drift region at x = 1.45 μm.

The two-dimensional electric field distribution when the device reaches its avalanche breakdown voltage is shown in Figure 4. At this time, the collector voltages of C-IGBT and H-IGBT are 15 and 17 kV, respectively. As can be seen from the figure, when the device reaches its breakdown voltage, the maximum internal electric fields of the conventional structure and proposed structure are 2.96 and 2.98 MV/cm, respectively. The breakdown voltage of the H-IGBT is larger than that of the C-IGBT because its hole injection efficiency is low, which is equivalent to lowering the doping concentration of the drift region. Therefore, when the device is blocked in the forward direction, the electric field strength of the entire drift region is raised, and the breakdown voltage of the H-IGBT is increased.

### 3.3. Turn-Off Characteristics

**Figure 5:**
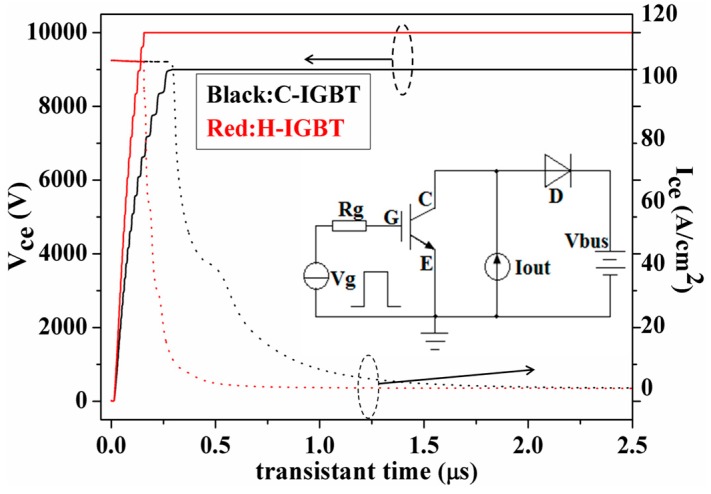
Turn-off characteristic curve and test circuit of C-IGBT and H-IGBT.

Since the device stores a large number of minority carriers in the drift region during the forward conduction process, the on-resistance is very small, but this is very disadvantageous for the turn-off process of the device. When the device is turned off, the carriers stored in the drift regions form a large tail current that extends the turn-off time of the device and greatly increases the power loss of the turn-off. In this simulation, we used the test circuit shown in Figure 5 to compare the shutdown performance of C-IGBT and H-IGBT. The clamped inductive load was modeled by a constant current source (2.1 × 10^-6^) and the bus voltage was set to 60% of the breakdown voltage. A gate voltage of 5 kHz, a 50% duty cycle, and a voltage change from 20 to –5 V were used to control the turn-on and turn-off of the device.

The turn-off characteristic curves of the conventional structure and the proposed structure are shown in Figure 5. It can be seen from the figure that the turn-off speed of the proposed structure is significantly better than that of the conventional structure, which is mainly due to the introduction of the p-polySi/p-SiC heterojunction on one side of the collector. After numerical calculation, it can be concluded that the turn-off losses of the traditional structure and the proposed structure were 7.7 and 3.2 mJ, respectively, and the turn-off loss was improved by about 58.4%. The reason why H-IGBT can have such excellent shutdown performance is mainly due to the introduction of a collector heterojunction. Through the adjustment of the doping concentration, the heterojunction can accelerate the extraction of electrons during the process of turning off the device, thereby reducing the turn-off loss. The above explanation can be further proven by the following analysis.

**Figure 6:**
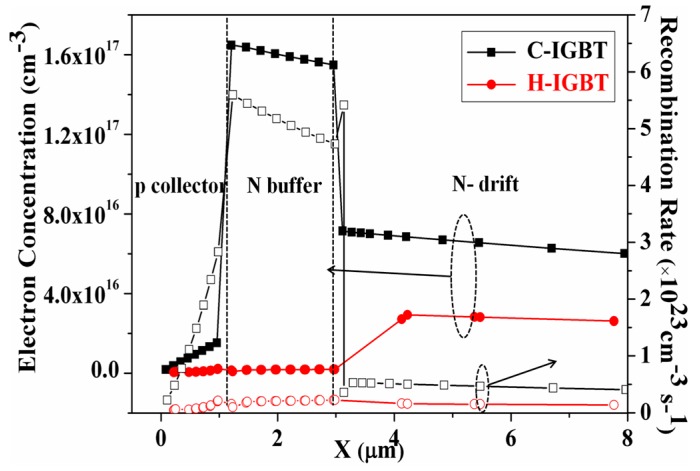
Electron concentration and carrier recombination rate on the collector side during voltage rise.

Figure 6 shows the electron concentration and carrier recombination rates near the collector of the conventional structure and the proposed structure during device turn-offs. It can be seen from the figure that the electron concentration and the carrier recombination rate of the conventional structure were higher than those of the proposed structure, indicating that the carriers generally disappeared by recombination when the conventional structure was turned off. The electron concentration and carrier recombination rate of the proposed structure were lower than those of the conventional structure, but the final turn-off loss was lower than that of the conventional structure, further indicating that the proposed structure had other bleed paths in addition to the composite bleed.

**Figure 7:**
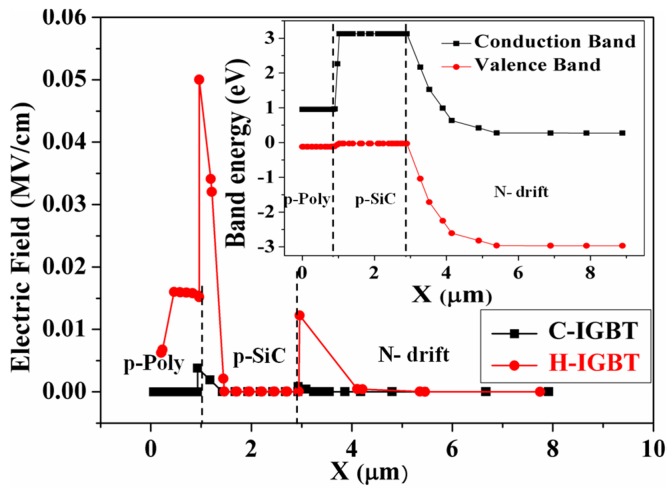
Electric field cut line and energy band diagram near the collector in the voltage rising phase.

The electric field stubs and the p-polySi/p-SiC heterojunction energy band diagram near the collector during device turn-offs is shown in Figure 7. As can be seen from the figure, when the proposed structure was turned off, a much larger electric field spike was introduced to the collector side than that of the conventional structure, and this larger electric field could drive more electrons from the drift region to the collector, thus accelerating the turn-off of the device. In addition, it can be seen from the energy band diagram of the heterojunction that the heterojunction portion was more favorable for electron bleed than the ordinary PN junction portion. Ultimately, the combined effect of the two bleeds mechanisms greatly reduced the turn-off losses of the proposed structure.

**Figure 8:**
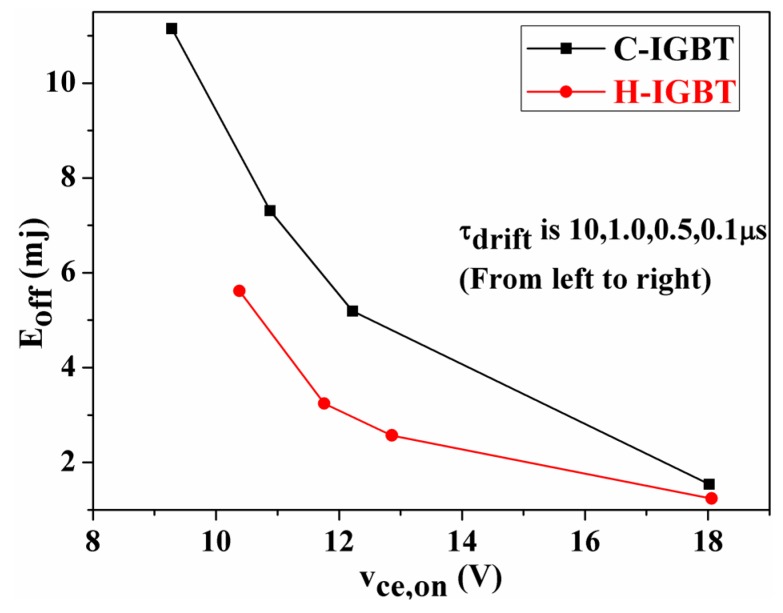
Tradeoff curve of conduction voltage drop and turn-off loss.

Figure 8 shows a compromise between the on-state voltage and turn-off loss of the device for different drift region carrier lifetimes. It can be seen from the figure that as the carrier lifetime in the drift region decreases, the turn-on voltage drop gradually increases and the turn-off loss gradually decreases. The main reason for this result is that the carrier lifetime injected into the drift region decreases as the carrier lifetime decreases, which weakens the positive conductance modulation effect and increases the on-state voltage drop. When the device is turned off, the carrier bleed time is reduced since less carriers are stored in the drift region, and the device turn-off loss is reduced.

## 4. Conclusions

We presented a H-IGBT with partial p-polySi/p-SiC heterojunction at the backside of the device, and compared the electrical characteristics of H-IGBT and C-IGBT with ATLAS simulation software. The simulation results showed that, under the appropriate doping concentration, the heterojunction part has little effect on the forward conduction characteristics, and at the same it can greatly improve the turn-off speed of the device. In the case of forward blocking, the electric field in the entire drift region of the device is raised due to the introduction of the heterojunction, so the breakdown voltage of the device is improved. Finally, compared with C-IGBT, the turn-off loss of H-IGBT is reduced by 58.4%, the breakdown voltage is increased by 13.3%, and the on-state voltage drop is increased by 8.3%.

## Figures and Tables

**Table 1:** Devices parameters for the simulations.

Parameter	C-IGBT	H-IGBT
Gate oxide wall thickness	0.05 μm	0.05 μm
Gate oxide bottom thickness	0.1 μm	0.1 μm
Half-cell width	2.1 μm	2.1 μm
N^-^ drift thickness	160 μm	160 μm
P^+^ source doping	5 × 10^19^ cm^−3^	5 × 10^19^ cm^−3^
N^+^ source doping	2 × 10^19^ cm^−3^	2 × 10^19^ cm^−3^
p-body doping	4 × 10^17^ cm^−3^	4 × 10^17^ cm^−3^
CSL doping	1 × 10^15^ cm^−3^	1 × 10^15^ cm^−3^
N^-^ drift doping	4.5 × 10^14^ cm^−3^	4.5 × 10^14^ cm^−3^
N buffer doping	1 × 10^17^ cm^−3^	1 × 10^17^ cm^−3^
P^+^ collector doping	1 × 10^19^ cm^−3^	1 × 10^19^ cm^−3^
p-SiC doping	—	1 × 10^19^ cm^−3^
p polysilicon doping P^+^ source region width N^+^ source region width p-SiC width p polysilicon width	— 1 μm 0.45 μm — —	1 × 10^17^ cm^−3^ 1 μm 0.45 μm 1.1 μm 1.1 μm
